# Roles of MAPK and Spindle Assembly Checkpoint in Spontaneous Activation and MIII Arrest of Rat Oocytes

**DOI:** 10.1371/journal.pone.0032044

**Published:** 2012-02-27

**Authors:** Wei Cui, Jie Zhang, Hua-Yu Lian, Hui-Li Wang, De-Qiang Miao, Chuan-Xin Zhang, Ming-Jiu Luo, Jing-He Tan

**Affiliations:** College of Animal Science and Veterinary Medicine, Shandong Agricultural University, Tai-an City, People's Republic of China; Ottawa Hospital Research Institute and University of Ottawa, Canada

## Abstract

Rat oocytes are well known to undergo spontaneous activation (SA) after leaving the oviduct, but the SA is abortive with oocytes being arrested in metaphase III (MIII) instead of forming pronuclei. This study was designed to investigate the mechanism causing SA and MIII arrest. Whereas few oocytes collected from SD rats at 13 h after hCG injection that showed 100% of mitogen-activated protein kinase (MAPK) activities activated spontaneously, all oocytes recovered 19 h post hCG with MAPK decreased to below 75% underwent SA during in vitro culture. During SA, MAPK first declined to below 45% and then increased again to 80%; the maturation-promoting factor (MPF) activity fluctuated similarly but always began to change ahead of the MAPK activity. In SA oocytes with 75% of MAPK activities, microtubules were disturbed with irregularly pulled chromosomes dispersed over the spindle and the spindle assembly checkpoint (SAC) was activated. When MAPK decreased to 45%, the spindle disintegrated and chromosomes surrounded by microtubules were scattered in the ooplasm. SA oocytes entered MIII and formed several spindle-like structures by 6 h of culture when the MAPK activity re-increased to above 80%. While SA oocytes showed one Ca^2+^ rise, Sr^2+^-activated oocytes showed several. Together, the results suggested that SA stimuli triggered SA in rat oocytes by inducing a premature MAPK inactivation, which led to disturbance of spindle microtubules. The microtubule disturbance impaired pulling of chromosomes to the spindle poles, caused spindle disintegration and activated SAC. The increased SAC activity reactivated MPF and thus MAPK, leading to MIII arrest.

## Introduction

The rat has been used as an important rodent model for physiological studies and for the analysis of multigenic human diseases such as hypertension, diabetes and neurological disorders [1]. The rat is important not only because it is larger than the mouse but also because a plethora of organ-specific physiologic and pathologic models have been developed for it in recent years [2]. Thus, intensive efforts have been made to establish the rat as a strong genetic animal model. However, many studies have failed to obtain rat offspring by somatic cell nuclear transfer [3]–[7]. Unlike oocytes from other animals, the rat oocytes undergo spontaneous activation (SA) soon after collection from the oviduct [8]. Somatic cell nuclei introduced into enucleated rat oocytes do not show premature chromosome condensation [4] and they might not be properly reprogrammed due to SA of the oocyte during manipulation for enucleation and introduction of somatic cells [9]. Inhibiting oocyte SA is thus of great importance for successful rat cloning by nuclear transfer.

In most mammals, matured oocytes are arrested at the metaphase II (MII) stage. This arrest is maintained by a high activity of the cytostatic factor (CSF) [10]. In Xenopus oocytes, Mos [11], p90rsk [12] and Emi1 [13] have been reported as the candidates for CSF. All these candidates are directly or indirectly involved in the inhibition of the anaphase promoting complexes/cyclosome (APC) which targets proteins like cyclin B and securin for degradation by the proteasome [14], [15]. In mice, it was reported that (i) oocytes derived from Mos-deficient mothers did not show the MII meiotic arrest [16], [17]; (ii) the p90rsk was associated with and phosphorylated Emi to induce the oocyte metaphase arrest [18]; and (iii) Emi2 was involved in both establishment and maintenance of the MII arrest [19]. In rat oocytes, treatment with MEK inhibitor (U0126) accelerated oocyte release from MII arrest and promoted pronuclear formation [20] suggesting that a high activity of Mos/MEK/MAPK is required for maintenance of the CSF activity. Because MAPK activation is regulated by the maturation-promoting factor (MPF) in the rat [21], and MPF inhibition induced pronuclear formation following inactivation of the MAPK pathway in mice [22], a high MPF activity may also be essential for the maintenance of CSF activity. However, although these data suggest that MPF and MAPK are candidates for CSF that maintain the MII arrest of rat oocytes, what downstream targets they regulate to induce SA are not known. Furthermore, after SA, rat oocytes do not form pronuclei but instead, they are arrested at the metaphase III (MIII) stage [23]. Freshly ovulated mouse oocytes were also arrested at the MIII stage after activation with ethanol treatment [24]. Although the MIII arrest in mouse oocytes was found to be associated with increased MPF activities [25], the mechanism causing the MIII arrest is largely unknown.

Previous studies demonstrated that whereas the Mos protein level and the activity of MEK/MAPK in SA oocytes from the Wistar rat decreased significantly at 2 h after collection, non-SA oocytes from the Sprague-Dawley (SD) rat showed a high level of Mos protein and MEK/MAPK activity [20]. In SA rat oocytes that were arrested at the MIII stage, normal spindles did not form but dispersed chromosomes surrounded by microtubules were observed [26]. Furthermore, it has been reported that MAPK is localized on the meiotic spindle [27]–[30] and that its activity is a critical regulator of microtubule assembly and spindle organization during oocyte maturation [30]–[32]. In addition, it was shown that defects in spindle assembly or spindle kinetochore attachment, or artificial depolymerization of microtubules, activated the spindle assembly checkpoint (SAC) proteins such as MAD2 and BUB1, which arrested cells prior to the metaphase-anaphase transition with stable cyclin B and elevated MPF activities [33], [34]. Further studies confirmed that a complex between APC, Cdc20 and SAC proteins renders APC inactive and thus activates MPF by preventing cyclin B proteolysis [35]–[38].

**Figure 1:**
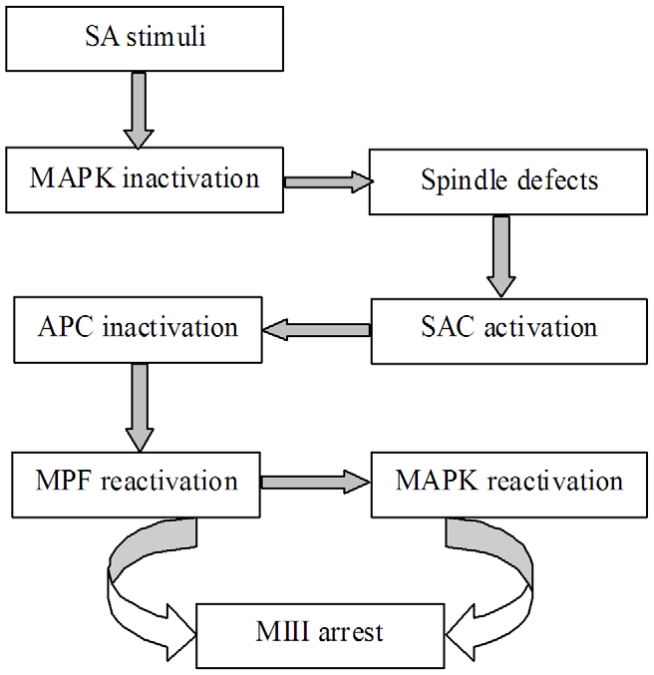
A diagram depicting the overall hypothesis of the study. Refer to the last paragraph of the Introduction section for detailed explanations.

We therefore hypothesized that the stimulus that triggers SA of rat oocytes would cause a premature inactivation of MAPK, which would impair spindle assembly and/or spindle kinetochore attachment (Fig. 1). The spindle defects would then activate SAC proteins, which reactivate MPF by inactivating APC. The increased MPF activity would then activate MAPK. High MPF and MAPK activities would trigger a return to M-phase (MIII arrest) of SA oocytes. To test this hypothesis, changes in chromosome spindles were first observed to see whether SA disturbs spindle assembly and to determine the timing of nuclear events during SA of rat oocytes. MPF and MAPK activities were then quantified, tested and correlated with changes in chromosome spindles to confirm that the spindle defects were caused by premature MAPK inactivation and that the return to MIII was triggered by reactivation of MPF and MAPK. Expression of SAC proteins and the effect of their neutralization were then observed to determine that spindle defects reactivated MPF by activating SAC. Ca^2+^ oscillations were finally examined to confirm our hypothesis that compared to sperm or chemical stimuli, SA was a weak activating stimulus that generated only a single Ca^2+^ rise and failed to activate APC. Changes after Sr^2+^-induced activation (IA) of newly ovulated rat oocytes were always observed in parallel for controls.

## Results

### Changes in chromosome spindles during SA or IA of rat oocytes

**Figure 2:**
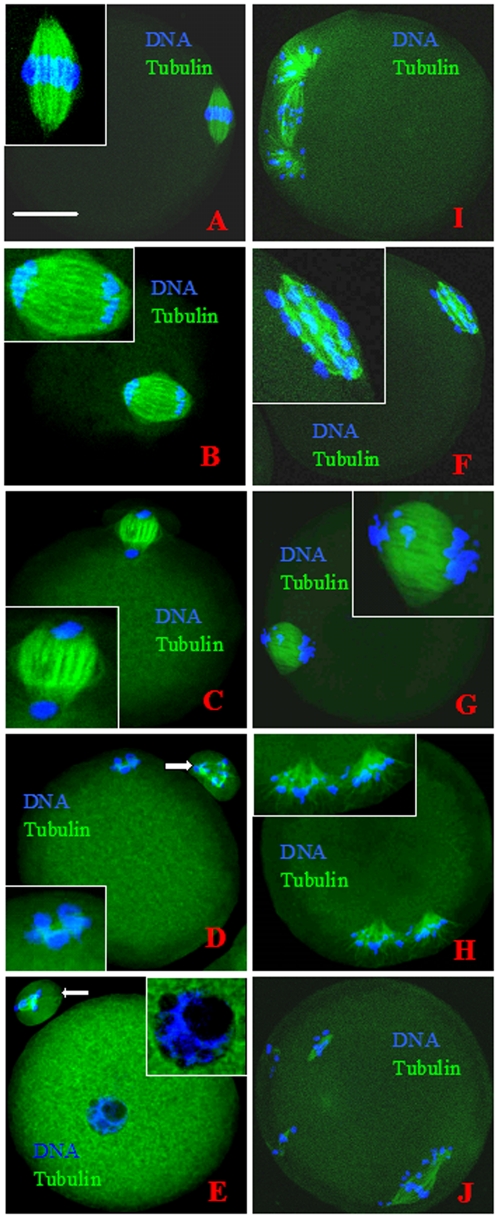
Confocal images of rat oocytes at different stages of IA (left column) or SA (right column). The DNA and α-tubulin in oocytes were pseudo-colored blue and green, respectively. A is an oocyte at the metaphase II (MII) stage showing a regular spindle with chromosomes aligned on the metaphase plate. B is an IA oocyte in anaphase II (AnII) showing a spindle with chromosomes tidily aligned on either pole. C is an IA oocyte in early telophase II (e-TelII) with a condensed chromosome mass on either pole of the spindle and the initiation of PB2 extrusion. D is an IA oocyte in late telophase II (l-TelII) with extruded PB2 and the initiation of chromosome decondensation. E is an IA oocyte in interphase (Int) with pronuclear formation. F is a SA oocyte in AnII with chromosomes dispersed over the surface of the spindle. G is a SA oocyte in e-TelII with chromosomes arranged toward the spindle poles. H and I are SA oocytes in l-TelII showing disintegrated spindles with chromosomes surrounded by microtubules scattered in the ooplasm. J is a SA oocyte at the MIII stage with microtubules reorganized into several small spindles around the scattered chromosomes. PB2 (arrow) was often observed in IA oocytes but not in SA oocytes. Scale bar is 20 µm.

This experiment was conducted to determine whether SA disturbs spindle assembly and the timing of nuclear events during SA of rat oocytes. To observe spindle changes during IA, freshly ovulated rat oocytes collected 13 h post hCG injection were treated with SrCl_2_ for IA. Oocytes were examined for spindle morphology at different times after Sr^2+^ treatment. All the freshly collected oocytes were at the MII stage (Table 1) showing a regular spindle with chromosomes aligned on the metaphase plate (Fig. 2A). At 0.5 h after Sr^2+^ treatment, most of the oocytes were in anaphase II (AnII) with sister chromatids tidily aligned at each pole of the spindle (Fig. 2B). At 1.5 h, most of the oocytes were in early telophase II (e-TelII) with chromosomes aggregated into a condensed mass at each pole of the spindle, often with the initiation of second polar body (PB2) extrusion (Fig. 2C). Most of the oocytes did not leave e-TelII until 3 h after Sr^2+^ treatment. By 6 h, most of the oocytes entered late telophase II (l-TelII) with extruded PB2 and initiating chromosome decondensation (Fig. 2D), whilst the rest entered the interphase (Int) with pronuclear formation (Fig. 2E).

**Table 1:** Percentages of oocytes at different stages of IA at different times following Sr^+^ treatment of rat oocytes collected 13 h post hCG injection.

Time of culture (h)	Oocytes observed	% MII oocytes	% Oocytes at different stages of IA				
			Total	AnII	e-TelII	l-TelII	Int
0	53	100^a^	0	0^a^	0^a^	0^a^	0^a^
0.5	58	42.0±5.4^b^	34	97.4±2.6^b^	2.6±2.6^a^	0^a^	0^a^
1	60	11.8±3.7^c^	53	44.8±13.6^c^	55.2±13.7^b^	0^a^	0^a^
1.5	57	0^d^	57	6.3±3.2^a^	93.7±3.2^c^	0^a^	0^a^
2	58	0^d^	58	0^a^	82.8±10.1^c^	17.2±10.1^b^	0^a^
3	58	0^d^	58	0^a^	61.1±5.6^b^	38.9±5.6^c^	0^a^
6	63	0^d^	63	4.0±2.1^a^	8.7±2.9^a^	67.1±6.9^d^	20.2±5.9^b^

a–d : Values with a common letter in their superscripts do not differ (P>0.05) in the same column. Each treatment was repeated 3–4 times with 15–20 oocytes in each replicate.

To observe spindle changes during SA, rat oocytes collected 19 h post hCG were aged for different times in mR1ECM before examination for spindle morphology. All the freshly collected oocytes were in MII (Table 2). At 0.5 h of in vitro aging, most of the oocytes entered AnII but with chromosomes dispersed throughout the surface of the spindle (Fig. 2F). At 1.5 h, whereas some oocytes were in e-TelII having a spindle with chromosomes arranged toward each pole (Fig. 2G), most reached l-TelII showing disintegrated spindles with chromosomes and microtubules scattered in the ooplasm (Fig. 2H and I). Most of the oocytes remained in l-TelII until 6 h of culture when 35% entered MIII with microtubules reorganized into several small chromosome spindles (Fig. 2J).

**Table 2:** Percentages of oocytes at different stages of SA at different times during culture in mR1ECM of rat oocytes collected 19 h post hCG injection.

Time of culture (h)	Oocytes observed	% MII oocytes	% Oocytes at different stages of SA				
			Total	AnII	e-TelII	l-TelII	MIII
0	50	100^a^	0	0^a^	0^a^	0^a^	0^a^
0.5	53	58.3±2.4^b^	22	86.9±7.2^b^	13.1±7.2^ab^	0^a^	0^a^
1	61	51.3±3.7^bc^	30	37.6±9.0^c^	52.9±9.3^c^	9.5±9.5^a^	0^a^
1.5	55	47.8±4.0^cd^	29	16.2±8.5^d^	23.6±8.2^b^	60.2±12. 6^b^	0^a^
2	64	47.6±4.0^cd^	34	0^a^	7.2±3.7^ab^	77.8±8.3^bc^	15.0±7.6^a^
3	65	40.8±2.0^d^	38	4.2±4.2^ad^	0^a^	82.1±5.4^c^	13.7±8.3^a^
6	45	48.6±3.9^cd^	23	0^a^	4.2±4.2^a^	61.3±6.2^bc^	34.5±7.8^b^

a–d : Values with a common letter in their superscripts do not differ (P>0.05) in the same column. Each treatment was repeated 3–4 times with 15–20 oocytes in each replicate.

### Changes in MPF and MAPK activities during IA and SA of rat oocytes

**Figure 3:**
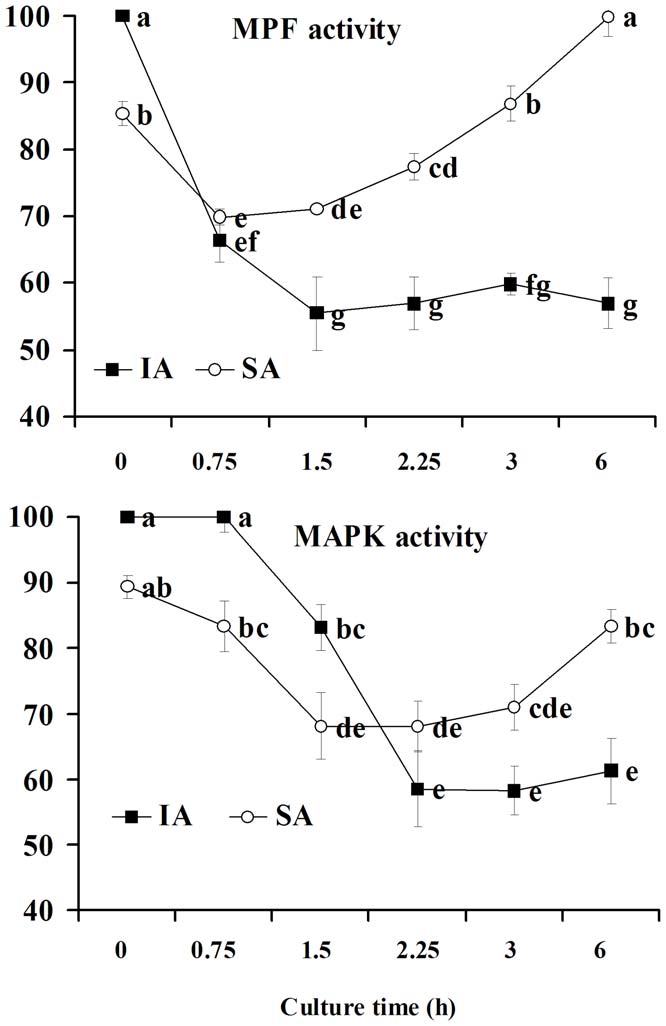
Changes in MPF and MAPK activities of rat oocytes during SA of in vitro culture or during IA after Sr^2+^ treatment. Oocytes for SA were collected 19 h post hCG and cultured for different times in mR1ECM medium before kinase assay. For IA, newly ovulated oocytes collected 13 h post hCG injection were treated with SrCl_2_ for 15 min and were assayed for kinase activities at different times after SrCl_2_ treatment. a-g: Values without a common letter differ (P<0.05).

The following experiments were designed to determine the dynamics of MPF and MAPK during oocyte SA to confirm that the spindle defects were caused by premature MAPK inactivation and that the MIII arrest was triggered by reactivation of MPF and MAPK. Newly ovulated oocytes collected 13 h after hCG administration were activated with SrCl_2_ and MPF and MAPK activities were assayed at different times after IA. Oocytes collected 19 h post hCG were cultured in mR1ECM for different times before assay for kinase activities during SA. Whereas freshly ovulated oocytes show 100% of MPF and MAPK activities, both kinase activities decreased to about 85% in oocytes recovered for SA 19 h post hCG (Fig. 3). During IA, the MPF activity decreased immediately after Sr^2+^ treatment and reached the lowest level by 1.5 h, but the MAPK activity did not decline until after 0.75 h and did not reach the lowest level until 2.25 h after Sr^2+^ treatment. During SA, however, both MPF and MAPK activities declined immediately after culture and reached the lowest level in close succession at 0.75 h and 1.5 h of culture, respectively. After that, while both kinase activities remained constant at the lowest level during IA, they went up significantly during SA to above their level at the onset of culture.

**Figure 4:**
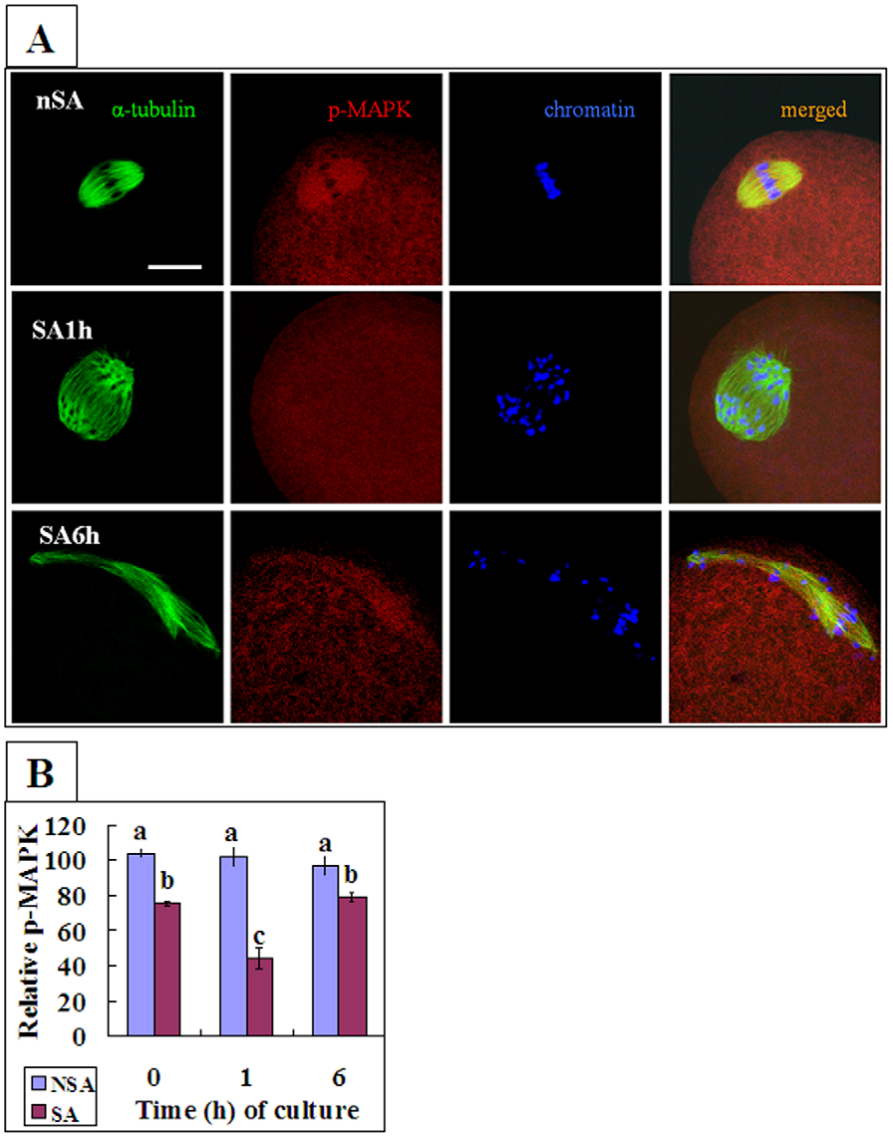
Distribution and quantification of phosphorylated MAPK (p-MAPK) in rat oocytes. Oocytes collected 19 h post hCG were aged for different times in mR1ECM before examination for p-MAPK expression. A. Laser confocal micrographs showing p-MAPK distribution. Whereas images in different rows show a non-SA oocyte remaining at the MII stage (nSA), a SA oocyte observed at 1 h (SA1h) and a SA oocyte observed at 6 h (SA6h) of in vitro aging, respectively, images in different columns show α-tubulin, p-MAPK, chromatin and merged pictures, respectively. The scale bar is 20 µm. B is a graph showing p-MAPK quantification in SA and non-SA (NSA) oocytes aged in vitro for 0, 1 and 6 h. Each treatment was repeated 3 times and each replicate contained 6–8 oocytes. Values without a common letter above their bars differ (p<0.05).

Because only about half of the oocytes underwent SA during in vitro aging, while almost all the oocytes underwent IA after Sr^2+^ treatment, we expected that the difference in MAPK activity between SA and IA oocytes would be more remarkable if the MAPK activity was detected in only those oocytes that had actually initiated SA. To test this expectation, rat oocytes collected 19 h post hCG were aged for different times in mR1ECM before examination for p-MAPK expression. At 0.5 h and 1 h of aging, whereas non-SA oocytes with tidily arranged spindle chromosomes showed marked expression of p-MAPK on their spindles (Fig. 4A, nSA), p-MAPK expression was either faint or undetectable in SA oocytes with dispersed spindle chromosomes (Fig. 4A, SA1h). By 6 h of aging, however, p-MAPK expression became marked again in SA oocytes arrested in MIII (Fig. 4A, SA6h).

The relative p-MAPK contents of SA oocytes were then quantified by measuring fluorescence intensities in confocal images. Oocytes collected 19 h post hCG were aged for 0, 1 and 6 h before p-MAPK quantification. Oocytes aged for 0 h were divided into those destined to undergo SA with less p-MAPK and those not destined with more p-MAPK. Oocytes aged for 1 or 6 h were classified as SA and non-SA according to morphology. The average fluorescence of total 0-h oocytes was set as 89.4% as measured in the MBP kinase assay (Fig. 3) and the averages of oocytes in other treatments were expressed relative to this value. Whereas the not-destined and non-SA oocytes showed about 100% of p-MAPK at different aging intervals, p-MAPK contents in the destined and SA oocytes first decreased but then increased again (Fig. 4B). Taken together, the results suggested that (i) during SA of rat oocytes, both MPF and MAPK ran an abortive decline with their activities increasing again before touching the same bottom as that observed during IA; (ii) the MPF activity began to change always ahead of the MAPK activity; and (iii) all the oocytes initiated SA when MAPK activity decreased to 75%.

### Down regulation of the MAPK activity with U0126 caused spindle disintegration and chromosome dispersion while suppressing PB2 extrusion in IA oocytes

To further confirm the role of MAPK activities in maintaining spindle integrity and PB2 emission after activation of rat oocytes, oocytes recovered 13 h after hCG were first treated with SrCl_2_ for 15 min in the presence of 50-µM U0126 and then incubated for 3 h in regular mR1ECM with U0126. Oocytes were then cultured for 3 h in mR1ECM without U0126. When examined at 1.5 h after Sr^2+^ treatment, whereas almost all (118/123) the control oocytes that had not been treated with U0126 showed a normal e-TelII spindle with a condensed chromosome mass on each pole and the initiation of PB2 extrusion, all (142/142) the oocytes treated with U0126 showed no PB2 extrusion but disintegrated spindles with chromosomes dispersed in the ooplasm. This verified that a premature MAPK inactivation was responsible for the disintegration of chromosome spindles and the failure of PB2 emission during SA of rat oocytes. However, some (69/142) of the U0126-treated oocytes formed pronuclei when observed at 6 h after Sr^2+^ treatment.

### Defects in spindle microtubules activated SAC during SA of rat oocytes

**Figure 5:**
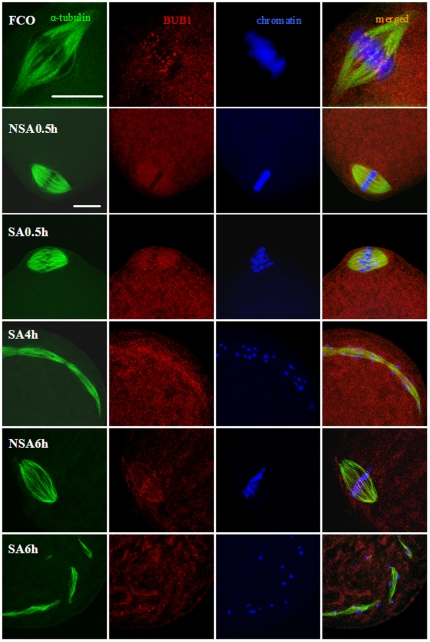
Laser confocal micrographs showing BUB1 in rat oocytes. Oocytes collected 19 h post hCG were aged for different times in mR1ECM before examination for BUB1. The α-tubulin, BUB1 and chromatin were pseudo-colored green, red and blue, respectively. Images in different rows show freshly collected oocyte in MII (FCO), non-SA oocytes observed at 0.5 h (NSA0.5h) and 6 h (NSA6h), and SA oocytes observed at 0.5 h (SA0.5h), 4 h (SA4h) and 6 h (SA6h) of in vitro aging, respectively. Images in different columns show α-tubulin, BUB1, chromatin and merged pictures, respectively. Scale bars are 15 µm.

Two experiments were conducted to test the hypothesis that spindle defects reactivated MPF by activating SAC. In the first experiment, oocytes collected 19 h after hCG were cultured in mR1ECM for different times before examination for distribution of BUB1. Whereas BUB1 was localized on individual chromosome kinetochores in oocytes examined immediately after collection (Fig. 5, FCO), BUB1 signals disappeared completely from kinetochores and became distributed on spindle microtubules in oocytes examined between 0.5 h and 4 h of culture, whether the oocytes were undergoing SA (Fig. 5, SA0.5 and SA4h) or not (Fig. 5, NSA0.5h). By 6 h of culture, however, BUB1 disappeared from spindle microtubules and localized again on chromosome kinetochores, whether oocytes were arrested at MII (Fig. 5, NSA6h) or MIII stage (Fig. 5, SA6h). The results suggested that whether rat oocytes underwent SA or not, (i) spindle microtubules were disturbed within 0.5 h after oocytes left the oviduct, (ii) the microtubule defects activated SAC and (iii) BUB1 underwent a kinetochore-microtubule-kinetochore translocation during in vitro aging.

In the second experiment, oocytes collected 19 h after hCG injection were cultured for aging in mR1ECM for 5 h before they were injected with either anti-MAD2 or anti-BUB1 antibodies or IgG. After injection, oocytes were cultured for 6 h in the KSOM medium before examined for pronuclear formation. Whereas only a few oocytes cultured without injection or injected with IgG formed pronuclei, about 65% of oocytes injected with anti-MAD2 or anti-BUB1 antibodies formed pronuclei (Table 3). The results strongly suggested that during SA of rat oocytes, defects in spindle microtubules reactivated MPF and MAPK and triggered MIII arrest by activating SAC.

**Table 3:** Pronuclear formation after rat oocytes were injected with BUB1 or MAD2 antibodies.

Injected with	Oocytes observed	% Oocytes with pronuclei
Nothing	109	0.6±0.6^a^
Rabbit IgG	83	20.9±3.6^b^
Rabbit anti-BUB1 antibodies	90	63.9±3.6^c^
Goat IgG	80	14.6±1.6^b^
Goat anti-MAD2 antibodies	83	62.1±5.0^c^

Rat oocytes collected 19 h post hCG were aged in mR1ECM for 5 h before antibody injection and after the injection, the oocytes were cultured for 6 h for pronuclear formation. Each treatment was repeated 3 times with about 25–35 oocytes in each replicate.

a–c : Values with different letter in superscripts differ significantly (P<0.05) within a column.

### Ca^2+^ oscillations during IA or SA of rat oocytes

**Figure 6:**
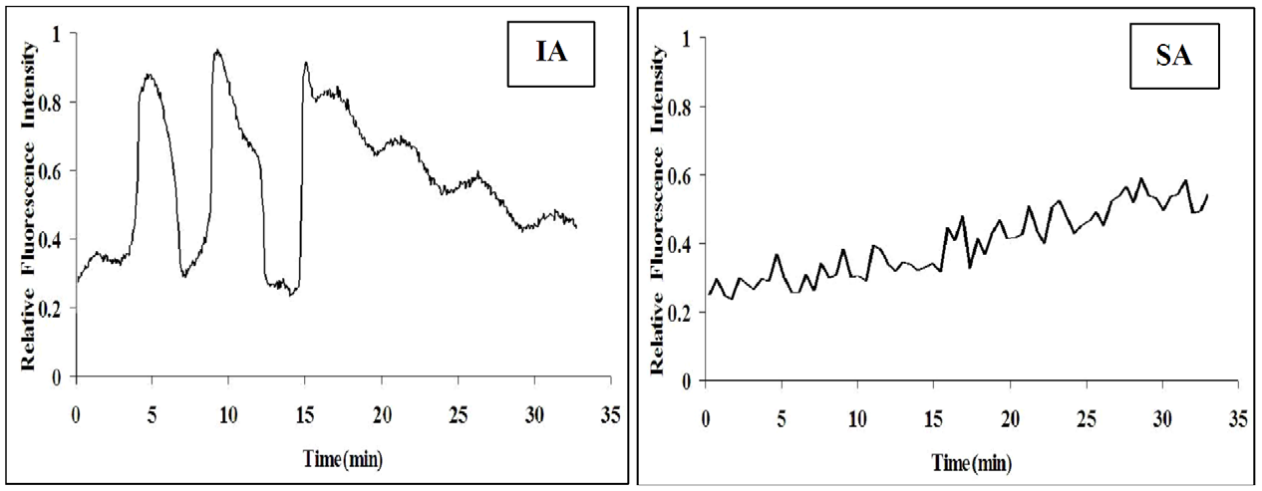
Ca^2+^ oscillations during IA or SA of rat oocytes.

To confirm our hypothesis that compared to sperm or chemical stimuli, SA was a weak activating stimulus that generated only a single Ca^2+^ rise that failed to activate APC, Ca^2+^ oscillations were measured during IA or SA of rat oocytes. To measure Ca^2+^ oscillations during IA, rat oocytes collected 13 h after hCG were treated with SrCl_2_ for 15 min and Ca^2+^ transients were measured for 35 min during and after Sr^2+^ treatment. To measure Ca^2+^ oscillations during SA, oocytes collected 19 h post hCG were measured for Ca^2+^ transients for 35 min during culture in aging medium. Results showed that IA oocytes showed 2–3 Ca^2+^ rises, but SA oocytes presented only a single slow Ca^2+^ increase (Fig. 6).

## Discussion

**Figure 7:**
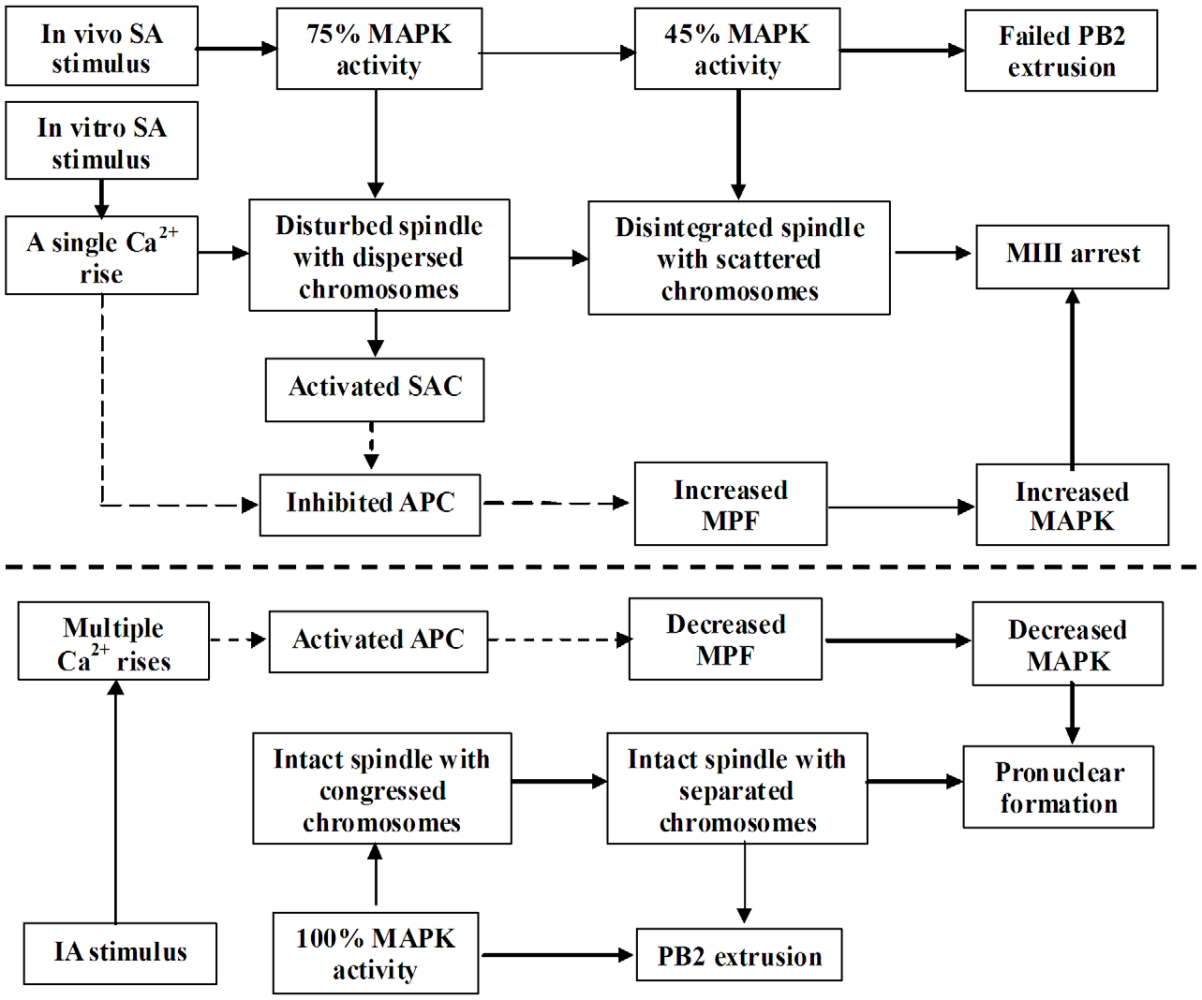
Possible pathways leading to the MIII arrest in SA oocytes and the pronuclear formation in IA oocytes. For a detailed explanation, please refer to the text in the Discussion section. Because APC activity was not actually examined in this study, dotted lines were used to depict the possible pathways involving the APC activity in this figure.

This study demonstrated for the first time that a premature MAPK inactivation triggered SA of rat oocytes by disturbing spindle integrity. Thus, whereas only 10% (14/137) of the oocytes collected 13 h after hCG injection that showed 100% of MAPK activities activated spontaneously after in vitro culture, all the oocytes recovered 19 h post hCG with MAPK activities decreased to 75% initiated SA with chromosomes dispersed on the perturbed spindle (Fig. 7). When MAPK activity decreased further to 45%, the spindle of SA oocytes disintegrated and chromosomes surrounded by microtubules became scattered in the ooplasm. Our results that down regulation of MAPK activity with U0126 caused spindle disintegration and chromosome dispersion in IA rat oocytes further highlighted the role of high MAPK activity in maintaining spindle integrity. It has been reported that MAPK is a critical regulator of microtubule assembly and spindle organization during maturation of mouse [30], [31] and rat oocytes [32]. A high activity of Mos/MEK/MAPK has been found to be essential for maintenance of the MII arrest in both mouse and rat oocytes. For example, mouse oocytes lacking the mos gene could not be arrested in MII but underwent SA immediately after maturation [16], [17], [39], due to a lack of active MEK and MAPK [40], [41]. Inhibition of MEK1/2 with U0126 parthenogenetically activated mouse eggs, producing phenotypes similar to Mos(−/−) parthenotes (premature, unequal cleavages and large polar bodies) [22]. Furthermore, treatment with U0126 accelerated rat oocyte release from MII arrest and pronuclear formation [20].

This study showed that the MIII arrest in SA rat oocytes was associated with MPF and MAPK reactivation. Freshly ovulated mouse MII oocytes were also arrested at the MIII stage after ethanol treatment [24]. During the MII/MIII transition of mouse oocytes, the MPF activity dropped at the time of PB2 extrusion and increased again in MIII oocytes [25]. However, it is unclear how MPF is activated during the MII/MIII transition. For the first time, the present results showed that the in vitro SA stimuli disturbed spindle microtubules in aged oocytes with MAPK activities down regulated by in vivo SA stimuli, and the defects in microtubules then activated MPF by activating SAC (Fig. 7). Thus, whereas BUB1 was localized on individual chromosome kinetochores in oocytes examined immediately after collection, BUB1 signals disappeared completely from kinetochores and became distributed on spindle microtubules in oocytes examined between 0.5 h and 4 h of culture, whether the oocytes were undergoing SA or not. By 6 h of culture, however, BUB1 disappeared from spindle microtubules and localized again on the chromosome kinetochores, whether the oocytes were arrested at MII or MIII stage. BUB1 is associated with kinetochores and is phosphorylated during the MII arrest of mouse oocytes [42]. Translocation of BUB1 from spindle poles to spindle microtubules was observed in mouse oocytes when the spindle was perturbed with nocodazole [43]. According to Yin et al. [43], translocation of BUB1 from spindle poles to spindle microtubules means that the damaged microtubules can attract BUB1 to strengthen the spindle, which in turn delays the onset of anaphase until Bub1 aids spindle recovery and chromosomes congregate properly at the metaphase plate. Since it has been reported that when not bound to kinetochores, BUB1 can still recruit substantial amounts of MAD1, MAD2 and MCAK to unattached kinetochores [44], we deduced that BUB1 on spindle microtubules could still function to activate MPF during SA of rat oocytes. To test this hypothesis, we injected BUB1 and MAD2 antibodies into in vitro aged rat oocytes before MIII arrest. Injection of either BUB1 or MAD2 antibodies increased pronuclear formation significantly. This strongly suggested that during SA of rat oocytes, defects in spindle microtubules activated MPF by activating SAC and that MAD2 was still active when BUB1 was localized on spindle microtubules instead of kinetochores. In addition, this study showed that the MPF activity began to change always ahead of the MAPK activity during SA of rat oocytes. It has been reported that MAPK activation is regulated by MPF in rat oocytes [21], and that MPF inhibition induced pronuclear formation following inactivation of the MAPK pathway in mouse oocytes [22].

In the present study, whereas rat oocytes formed well-developed pronuclei after IA, they were arrested in MIII after SA. Likewise, whereas freshly ovulated mouse oocytes formed pronuclei following Sr^2+^ treatment for 6 h (unpublished data of this laboratory), they were arrested in MIII after treatment with ethanol for 6.5 min [24]. It is well known that sperm activate oocytes by inducing a series of Ca^2+^ spikes that last for several hours. The Ca^2+^ signal causes activation of APC, leading to the destruction of key proteins necessary for meiotic arrest [45]. Calmodulin-dependent protein kinase II (CaMKII) activities increased during fertilization [46]. CaMKII was found to be sufficient for triggering cell-cycle resumption in mouse eggs and to act downstream of sperm-induced Ca^2+^ release but upstream of a spindle checkpoint [47]. In a model proposed by Jones [48], sperm-specific phospholipase C generates Ca^2+^ spikes to activate CaMKII and so switch on APC. Since it was found that multiple Ca^2+^ spikes were needed for continued cyclin degradation [49], we proposed that the difference between SA and IA rat oocytes in their ability to form pronuclei might be related to the strength of the activating stimuli. Thus, whereas the IA stimulus was strong enough to generate multiple Ca^2+^ rises that could activate APC adequately to promote pronuclear formation, the SA stimulus was weak to induce only a single Ca^2+^ rise that could not activate APC that was inhibited by activated SAC. The hypothesis was well confirmed by our results that whereas Sr^2+^ treatment for 15 min induced several intracellular Ca^2+^ increases in IA oocytes, oocytes cultured for SA showed only a single slow Ca^2+^ increase. Similarly, it was shown in mouse oocytes that whereas repetitive intracellular Ca^2+^ increases were induced following a 40 min Sr^2+^ treatment, only a single Ca^2+^ rise was observed after ethanol or Sr^2+^ treatment for 5 min [50]. The hypothesis was further supported by our results that some of the IA oocytes formed pronuclei even though their microtubules had been disturbed after U0126 treatment.

Whereas the ethanol-activated mouse oocytes extruded PB2 before MIII arrest [24], SA rat oocytes in this study were arrested at MIII without PB2 emission (Fig. 7). Although previous studies also observed MIII arrest without PB2 emission in SA rat oocytes [8], [23], [51], the mechanism is unclear. During the MII-MIII transition of the ethanol-activated mouse oocytes, although the MPF activity first dropped to the bottom level at the time of PB2 extrusion and then increased again in MIII oocytes, the MAPK activity remained high [25]. This dynamics of the kinases is different from what the present study observed during the MII-MIII transition of rat SA oocytes, where both kinase activities decreased significantly soon after activation and then both increased again. In their SA rat oocytes that showed high rates of PB2 emission, Yoo and Smith [52] observed no significant decrease in the MAPK activity although the MPF activity decreased significantly. Furthermore, it seems that oocytes recovered earlier from the oviduct (15–17 h after hCG injection) tend to be arrested at MIII after PB2 extrusion [23], [24], [53], whilst those recovered later (19 h after hCG) tend to be arrested without PB2 extrusion ([51] and the present study). It could be that when the MPF activity decreased to a level sufficient to allow PB2 extrusion immediately after culture, the MAPK level in young oocytes was still high enough but that in aged oocytes had decreased to below the threshold to support PB2 emission. Our results that down regulating MAPK activity with U0126 suppressed PB2 extrusion of IA oocytes provided further evidence for this expectation.

In summary, for the first time, we have conducted a systematic study on the mechanism causing SA and MIII arrest of rat oocytes. Results indicated that in the in vivo aged oocytes recovered 19 h post hCG injection, the in vitro SA stimulus (manipulation at room temperature and/or culture in vitro) was weak to induce only a single Ca^2+^ rise that disturbed spindle microtubules leading to dispersion of the irregularly-pulled chromosomes on the spindle, because the MAPK activity in these oocytes had been down regulated to 75% by the in vivo SA stimulus and was not enough to maintain microtubule integrity (Fig. 7). During aging in vitro, the MAPK activity continued to decrease to 45% as the MPF declined further. The decrease of MAPK to 45% resulted in (i) disintegration of the chromosome spindle with chromosomes scattered in the ooplasm and (ii) suppression of PB2 extrusion. Although 75% of MAPK activity was not enough to maintain microtubule integrity, it was enough to support SAC activation. As a result, a disturbance of spindle microtubules activated SAC. Because a single Ca^2+^ rise would not be enough to overcome the suppression of SAC on APC, the activated SAC would inhibit APC and increase MPF activity which then activated MAPK, leading to the MIII arrest. When freshly ovulated oocytes recovered 13 h post hCG were Sr^2+^ activated, their 100% MAPK activity maintained both spindle integrity and PB2 extrusion. Meanwhile, the Sr^2+^ stimulation generated multiple Ca^2+^ spikes that could activate APC adequately to inactivate MPF and MAPK and promoted pronuclear formation. In conclusion, we report for the first time that (i) a premature MAPK inactivation triggered SA (dispersion of chromosomes) by disturbing spindle microtubules and caused PB2 retention during SA of rat oocytes; (ii) the microtubule disturbance reactivated MPF by activating SAC, triggering the MIII arrest; and (iii) the SA stimulus induced only a single Ca^2+^ rise, whereas Sr^2+^ treatment induced multiple Ca^2+^ increases in rat oocytes.

## Materials and Methods

### Ethics Statement

Rat care and use were conducted exactly in accordance with the guidelines and approved by the Animal Research Committee of the Shandong Agricultural University, P. R. China (Permit number: 20010510). According to the guidelines of the committee, the animal handling staff (including each post-doc, doctor or master student) must be trained before using animals. Rats must be housed in a temperature-controlled room with proper darkness-light cycles, fed with a regular diet, and maintained under the care of the Experimental Animal Center, Shandong Agricultural University College of Animal Science and Vet Medicine. In the present study, Rats were sacrificed by cervical dislocation. The only procedure performed on the dead animals was the collection of oocytes from the oviducts.

Chemicals and reagents used in this study were purchased from Sigma Chemical Co. (St. Louis, MO, USA) unless otherwise specified.

### Oocyte recovery

Sprague-Dawley (SD) rats were kept in an air-conditioned room with 14 h/10 h light-dark cycles, the darkness starting from 8 pm. Female rats, 23–26 days after birth, were induced to superovulate with equine chorionic gonadotropin (eCG, 15 IU, ip) followed 48 h later by human chorionic gonadotropin (hCG, 15 IU, ip). Both eCG and hCG used in this study were from Ningbo Hormone Product Co., Ltd, P. R. China. The superovulated rats were sacrificed at different times after hCG injection and the oviductal ampullae were broken to release oocytes. After dispersed and washed three times in M2 medium, the oocytes were denuded of cumulus cells by pipetting with a thin pipette in a drop of M2 containing 0.1% hyaluronidase.

### Oocyte aging *in vitro*

To observe SA, rat oocytes recovered 19 h post hCG were cultured for different times in the modified rat 1-cell embryo culture medium (mR1ECM) [54], [55]. The aging culture was conducted in wells (20–35 oocytes per well) of a 96-well culture plate containing 200 µl of mR1ECM covered with mineral oil at 37°C under 5% CO_2_ in humidified air.

### Induced activation (IA)

To artificially induce activation, freshly ovulated oocytes recovered 13 h post hCG injection were treated for 15 min with 2 mM SrCl_2_ contained in Ca^2+^-free mR1ECM. After the activation treatment, the oocytes were cultured in regular mR1ECM for different times before other observations.

### Immunofluorescence microscopy

All the procedures were conducted at room temperature unless otherwise specified. Oocytes were washed 3 times in M2 between treatments. Oocytes were (i) fixed with 3.7% paraformaldehyde in PHEM buffer (60 mM Pipes, 25 mM Hepes, 10 mM EGTA and 4 mM MgSO_4_, pH 7.0) for 30 min at 37°C, followed by treatment with 0.5% Triton-X 100 in PHEM for 15 min; (ii) blocked in PHEM containing 3% BSA for 1 h; (iii) incubated overnight with rabbit anti-p-ERK1/2 (1∶500, Cell Signaling Technology, Beverly, MA) or rabbit anti-Bub1 (1∶500, Abcam, Cambridge, MA) in 3% BSA in M2 at 4°C; (iv) incubated for 1 h with Cy3-conjugated goat-anti-rabbit IgG (1∶800, Jackson ImmunoResearch) in 3% BSA in M2; (v) incubated for 1 h with fluorescein isothiocyanate (FITC)-conjugated anti-α-tubulin monoclonal antibody (1∶50) in 3% BSA in M2; (vi) incubated for 10 min with 10 µg/ml Hoechst 33342 in M2. To observe chromosome spindles, blocked oocytes were subjected to procedures v and vi only to stain tubulin and chromosomes, respectively.

The stained oocytes were mounted on glass slides and observed with a Leica laser scanning confocal microscope (TCS SP2; Leica Microsystems). Blue diode (405 nm), argon (Ar; 488 nm) and helium/neon (He/Ne; 543 nm) lasers were used to excite Hoechst, FITC and Cy3, respectively. Fluorescence was detected with the following bandpass emission filters: 420–480 nm (Hoechst), 505–540 nm (FITC) and 560–605 nm (Cy3), and the captured signals were recorded as blue, green and red, respectively. The individual optical sections were digitally recombined into a single composite image using the Leica Confocal Software.

The relative content of p-MAPK was quantified by measuring fluorescence intensities. For each experimental series, all images were acquired with identical settings. The relative intensities were measured on the raw images using Image-Pro Plus software (Media Cybernetics Inc., Silver Spring, MD) under fixed thresholds across all slides.

### Assay of H1 and MBP kinase activities

The H1 and MBP kinase activities were measured as follows. Forty cumulus-free oocytes from each treatment were washed three times in the histone kinase buffer (15 mM 3-[N-Morpholino] propanesulfonic acid [MOPS], pH 7.2, containing 80 mM β-glycerophosphate, 10 mM EGTA, 15 mM MgCl2, 0.1 mM PMSF, 10 µg/ml leupeptin, 10 µg/ml aprotinin, and 10 µg/ml cAMP-dependent protein kinase inhibitor peptide), transferred to 10 µl histone kinase buffer contained in a 1.5 ml microfuge tube, and stored frozen at −70°C. Before kinase reactions, the frozen samples were subjected to 4–5 times freezing and thawing to prepare lysates. Kinase reactions were initiated by the addition of 10 µl of substrate buffer containing 2 mg/ml histone H1, 2 mM dithiothreitol and 5 µCi [γ-32P] ATP to each sample, and the reactions were carried out for 50 minutes at 37°C. The reaction was terminated by the addition of an equal volume of double-strength SDS sample buffer containing β-mercaptoethanol, and the mixture was boiled for 3–5 minutes. Kinase reaction products were then separated by 12% linear gradient SDS-PAGE. Gels were exposed to phosphor-screens. Data acquisition was the actual scanning of sample images with the Cyclone® Plus Storage Phosphor System to create an image file that can be analyzed by the OptiQuant™ Image Analysis Software. The H1 kinase activity values of newly ovulated oocytes (recovered 13 h post hCG injection) were arbitrarily set as 100%, and the other values were expressed relative to this activity. The amount of kinase reaction product used for SDS-PAGE was strictly controlled (20 µl) for each sample, and three samples were analyzed for each treatment. The same procedures were repeated for assay of MBP kinase activity except that 2 mg/ml histone H1 was replaced with 1 mg/ml bovine myelin basic protein (MBP) in the substrate buffer.

### Microinjection of anti-BUB1 and anti-MAD2 antibodies

Microinjection of anti-BUB1 or anti-MAD2 antibodies was performed in HCZB medium using a Leica inverted microscope equipped with a piezo-driven micromanipulator. A volume of 3–5 pl of anti-BUB1 antibody (Abcam, Cambridge, MA, 1∶10 diluted in PBS) or anti-MAD2 antibody (Santa Cruz, 0.2 mg/ml in PBS) was injected into the cytoplasm of rat oocyte. The same amount of rabbit or goat IgG (0.2 mg/ml in PBS, Beyotime) was injected for controls. Each treatment was repeated three times, and about 35 oocytes were injected each time. All microinjections were completed within 30 min. After microinjection, oocytes were washed with M2 and cultured for 6 h in KSOM medium for examination of pronuclear formation.

### Calcium measurement

Intracellular Ca^2+^ was measured using the Ca^2+^-sensitive dye fluo-3. For loading, oocytes were incubated for 20 min at 37°C with 30 µM of the acetoxymethyl (AM) form of the dye made up in mR1ECM with 0.02% pluronic F-127. Drops of calcium-free or regular mR1ECM were made under paraffin oil in a Fluoro dish (World Precision Instruments, Inc.) with its base coated with phytoagglutinin. The drops were equilibrated overnight in a CO2 incubator. After loading, oocytes were washed with M2 and placed in the drops and the dish was transferred to a heated stage (37°C) of a Leica laser-scanning confocal microscope (TCS SP2; Leica Microsystems). To measure Ca^2+^ oscillations in IA oocytes, oocytes collected 13 h post hCG were placed in drops of calcium-free mR1ECM and SrCl_2_ was injected into the drops to produce a final concentration of 2 mM. At 15 min after SrCl_2_ injection, the Sr^2+^ treatment was terminated by diluting the drop with regular M2. Oocytes collected 19 h after hCG were placed in drops of regular mR1ECM for measurement of calcium oscillations in SA oocytes. An argon laser was used for excitation at 488 nm and signals emitted at 505–540 nm were collected by the laser scanning confocal imaging system. Traces of calcium oscillations were plotted using SigmaPlot 2000 software.

### Data analysis

There were at least three replicates for each treatment. Percentage data were arc-sine transformed and analyzed with ANOVA; a test of Duncan multiple comparisons was used to locate differences. The software used was Statistics Package for Social Science (SPSS 11.5; SPSS Inc., Chicago, IL, USA). Data were expressed as mean ± S.E.M. and P<0.05 was considered significant.
